# *Trametes versicolor* carboxylate reductase uncovered

**DOI:** 10.1007/s00706-016-1676-z

**Published:** 2016-03-01

**Authors:** Margit Winkler, Christoph K. Winkler

**Affiliations:** Institute of Molecular Biotechnology, Graz University of Technology, Graz, Austria; Department of Chemistry, Organic and Bioorganic Chemistry, University of Graz, Graz, Austria

**Keywords:** Bioreduction, Carboxylate reductase, Carboxylic acids, Aldehydes, Enzymes

## Abstract

**Abstract:**

The first carboxylate reductase from *Trametes versicolor* was identified, cloned, and expressed in *Escherichia coli*. The enzyme reduces aromatic acids such as benzoic acid and derivatives, cinnamic acid, and 3-phenylpropanoic acid, but also aliphatic acids such as octanoic acid are reduced.

**Graphical abstract:**

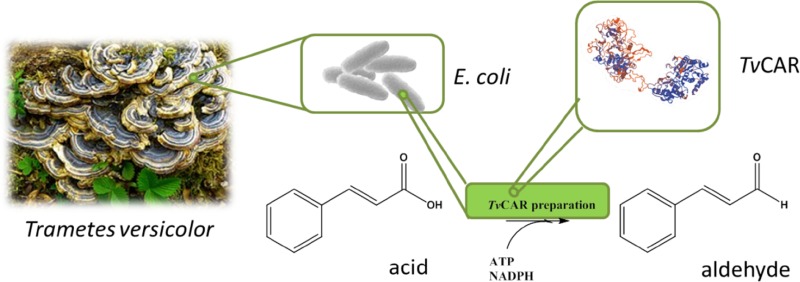

## Introduction

The reduction of carboxylic acids is a challenge both for chemical transformations and biotransformations. From the thermodynamic viewpoint, the carboxylic acid moiety is in an energetically favored state, shows little reactivity and needs a high level of activation to participate in chemical reactions. Due to the high stability of carboxylates, examples for their direct reduction are rare and usually an initial activation step (e.g., to the carboxylic ester or to the acyl halide) is required. The reduction of the COOH group or its derivatives yields the respective aldehyde in the first step; because aldehyde is more reactive than the carboxylic acid, the aldehyde does not accumulate but is typically reduced further to the respective primary alcohol. Certain reaction conditions may promote the accumulation of aldehydes instead of alcohol: the well-known examples are the reduction of acid chloride with lithium tri-*t*-butoxy aluminum hydride [1] or DIBALH [2]. In the past two decades, several selective chemical reductions to alcohols or aldehydes using hydrosilanes have also been described [3]. In laboratory practice, however, the acid is often fully reduced to the alcohol and then re-oxidized to the aldehyde [4], e.g., via the classical Swern or Dess–Martin oxidation [5]. Other oxidation protocols use chromate or manganese reagents, but also a large variety of methods using molecular oxygen in combination with immobilized transition metal catalysts have recently been developed [6]. For example, ruthenium or platinum on carbon in organic solvents serves as the catalyst for the oxidation of aromatic alcohols to the corresponding aldehydes [7, 8]. The use of organic solvents, toxic reagents, undesired reaction conditions or the high demand of reducing equivalents, however, induces a strong wish for one step alternatives to the selective preparation of aldehydes from acids.

A green substitution to chemical methods is the use of biocatalysts with carboxylic acid reduction ability. The first biocatalyzed reduction of carboxylic acid substrates was accomplished by the white-rot fungus *Trametes versicolor* [9]. A significant number of subsequent reports followed that described the reduction of carboxylic acids also by other fungi such as *Fomes fomentarius* [10], *Pycnoporus cinnabarinus* [11], *Aspergillus* sp. [11, 12], *Bjerkandera* sp. [13], *Mucor* sp. [14], and many others [15, 16]. However, to date, the amino acid sequence of only one single fungal carboxylate reductase has been elucidated from *Aspergillus terreus* [17, 18]. Herein, the sequence and substrate scope of the first carboxylate reductase from *T. versicolor* have been explored.

## Results and discussion

*Trametes versicolor* (alias: *Polystictus versicolor*) was reported to reduce benzoic acid derivatives to mixtures of aldehydes and alcohols [9]. The enzyme responsible for the first reduction step from the acid to the aldehyde had not been identified to this date, nor has a particular enzyme been purified and characterized. To discover the enzymatic activity on sequence level, the fungal carboxylate reductase enzyme sequence and three sequences of homologous bacterial ATP and NADPH-dependent carboxylate reductases were used as templates to search within the non-redundant protein sequences on NCBI with the restriction to the organism *T. versicolor*. The hits from this search were all annotated as acetyl-CoA synthetase-like proteins. *Aspergillus terreus* ATEG_03630 [17] is classified as CaiC for the adenylation domain and Thioester-redct for the reduction domain by the multi-domain model, whereas the CARs from *Nocardia iowensis* [19], *Mycobacterium marinum* [20], and *Segniliparus rotundus* [21] are classified as FAA1 and Lys2B. From the most significant hits of the search, one sequence was classified to consist of an FAA1 adenylation domain in combination with a Thioester-reductase domain. Therefore, it seemed most likely that this sequence would display carboxylate reductase activity.

Since homologous bacterial carboxylate reductases were shown to require the attachment of a phosphopantethein moiety [22], the protein was expressed from the pETDUET1 vector that harbored simultaneously *Escherichia coli* phosphopantethein transferase for post-translational modification of the carboxylate reductase and the coding sequence of the putative CAR enzyme with an *N*-terminal fusion tag. Despite codon optimization of the fungal gene sequence for expression in *E. coli*, the amount of soluble protein was disappointingly low and the majority of the protein was found in the insoluble fraction. The protein was purified via affinity chromatography, yielding *T. versicolor* CAR (*Tv*CAR) in enriched form. As known CAR enzymes have been well expressed heterologously in *E. coli* in their apo-form [22], the reason is unlikely insufficient post-translational phosphopantetheinylation. Optimization of the expression conditions, such as the choice of a weaker promoter, the use of chaperones [23], or ultimately a fungal expression system, will likely result in significant improvements with respect to enzyme yield.

In comparison to published CAR enzymes, *T. versicolor* CAR is most similar to *Aspergillus terreus* CAR, albeit with only 24 % identity according to UniProtAlign, whereas *N. iowensis* CAR and *M. marinum* CAR exhibit 17 % and *S. rotundus* CAR 16 % identity, respectively.

Carboxylate reductase enzymes rely on ATP and NADPH as cosubstrates. As typical for adenylating enzymes, the presence of Mg^2+^ is important due to its interaction with ATP [24]. *Tv*CAR preparation was assayed using the conditions as described in the experimental section. In addition, control reactions without the addition of substrate (*E*)-cinnamic acid (**1a**), ATP, or MgCl_2_ were carried out. NADPH was not oxidized in the absence of ATP or substrate. When the reaction was carried out in TrisHCl buffer without MgCl_2_, the carboxylate reduction rate was diminished by approximately 30 %. It needs to be noted that the enzyme preparation contained 10 mM MgCl_2_, resulting in 0.5 mM MgCl_2_ end concentration in this particular reaction.

*Tv*CAR was subjected to an NADPH depletion assay in the presence of different carboxylic acids. Enzymatic activity was observed for the substrates listed in Table 1. In the presence of, e.g. phenylacetic acid, mandelic acid, 2-pyrrole-, 2-pyridine-, 3-indole-, and 2-pyrazinecarboxylic acid as well as short chain aliphatic acids levulinic acid, pentanoic acid and hexanoic acid, NADPH was not oxidized by *Tv*CAR. The fact that **1a**, benzoic acid (**2a**), 3-hydroxy- and 3-methoxybenzoic acid (**3a**, **4a**) as well as 4-methoxybenzoic acid (**5a**) are reduced, but phenylacetic acid is not, is consistent with the observations of Farmer et al. [9]. However, 2-hydroxy- and 2-methoxybenzoic acids were not reduced by *Tv*CAR under the conditions used. This supports the idea that *T. versicolor* harbors more than one CAR enzyme and CAR (XP_008043822.1) is partly but not fully responsible for the reactions observed in 1959. As an analog of **1a**, 3-phenylpropanoic acid (**6a**) was also reduced. Surprisingly, also aliphatic carboxylates from C7 to C9 (**7a**–**9a**) were converted, whereas short chain acids and longer chain acids were not accepted as substrates under these conditions (Table 1).

**Table 1:** Substrate scope of *Trametes versicolor* carboxylate reductase

Entry	Substrate	Specific activity^a^/mU mg^−1^
1	(*E*)-Cinnamic acid (**1a**)	41 ± 4
2	Benzoic acid (**2a**)	30 ± 9
3	3-Hydroxybenzoic acid (**3a**)	22 ± 8
4	3-Methoxybenzoic acid (**4a**)	23 ± 5
5	4-Methoxybenzoic acid (**5a**)	33 ± 2
6	3-Phenylpropanoic acid (**6a**)	34 ± 7
7	Heptanoic acid (**7a**)	34 ± 7
8	Octanoic acid (**8a**)	35 ± 4
9	Nonanoic acid (**9a**)	33 ± 1

^a^One activity unit is defined as the amount of enzyme preparation catalyzing the oxidation of 1 µM of NADPH per minute

To investigate the identity of the reduction product, (*E*)-cinnamic acid (**1a**) was subjected to a biotransformation reaction in the presence of the *Tv*CAR enzyme and excess of co-factors. The reaction was analyzed after extraction and derivatization of the remaining substrate **1a** by GC/MS analysis, revealing cinnamaldehyde (**1b**) as the sole product in a mixture with methyl cinnamate (**1d**) (Scheme 1).

**Figure:**
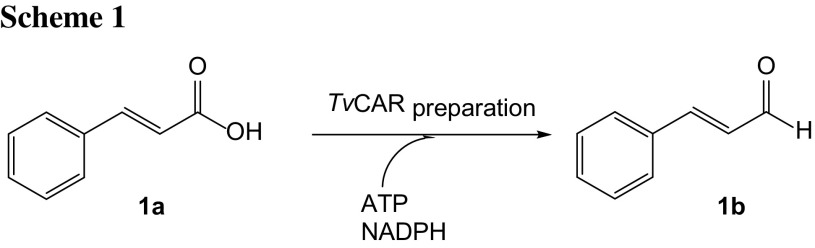

## Conclusion

The currently available portfolio of carboxylate reductase enzymes is limited to a handful of protein sequences. With the aim to enlighten new biocatalysts, we identified a putative acetyl-CoA synthetase-like protein from *T. versicolor*, expressed it in *E. coli* and confirmed that this protein indeed shows carboxylate reductase activity. The enzyme exhibits a promising substrate scope; however, its heterologous expression is a clear limitation at the moment.

### Experimental

Substrates, product references, DTT, and trimethylsilyldiazomethane (solution, 2 M in diethyl ether) were purchased from Sigma Aldrich and used without further purification. ATP was obtained from Roche Diagnostics. NADPH and MES were purchased from Roth, IPTG from Serva, and MgCl_2_ from Merck.

#### Cloning and expression

Four literature known carboxylate reductase enzyme sequences (Q6RKB1.1, WP_012393886.1, WP_013138593.1, and XP_001212808.1) were used as search templates for a blastp search against non-redundant protein sequences with the restriction to the organism *T. versicolor* (taxid:5325). A gene, coding for the protein sequence of NCBI accession code XP_008043822.1 was ordered as synthetic gene with optimization of the genetic code for expression in *E. coli*. The protein was expressed from the pETDUET1 vector with the *E. coli* pptase (NCBI accession code CAQ31055.1) cloned into the first multiple cloning site and *N*-terminally HIS-tagged CAR sequences in the second multiple cloning site. The plasmid was transfected to *E. coli* BL32(DE3) Star and colonies selected on LB/Amp. The enzyme was expressed using standard conditions. The cells were harvested by centrifugation and stored at −20 °C. After thawing, the cells were disrupted by sonication and the protein purified by nickel affinity chromatography, using the gravity flow protocol. The protein containing fractions were pooled and dialyzed overnight at 4 °C into 50 mM MES buffer, pH 7.5, containing 10 mM MgCl_2_, 1 mM EDTA, and 1 mM DTT. Aliquots of the resultant, slightly turbid protein solution were shock frozen in liquid nitrogen and stored at −80 °C.

#### Substrate screening

The capability of the protein to reduce carboxylic acids was determined using a photometric NADPH depletion assay. Therefore, potential carboxylic acid substrates were dissolved in KOH (0.1 M in water) or DMSO. The assay composition was as follows: The substrates (10 mm^3^ of 100 mM stock solution) were added to 160 mm^3^ of TrisHCl buffer (100 mM, pH 7.5, containing 10 mM MgCl_2_). Subsequently, 10 mm^3^ of NADPH (10 mM in water), 10 mm^3^ of ATP (20 mM in water), and 10 mm^3^ of enzyme preparation (3.85 mg/cm^3^ with an estimated purity of 50 %) were added. Each reaction was measured four times in parallel in UV-Star 96 well plates (Greiner). The depletion of NADPH was followed on a Synergy Mx Platereader at 340 nm and 28 °C for 10 min. Blank reactions without enzyme preparation were carried out in parallel.

#### Biotransformation

**1a** (10 mm^3^ of 100 mM stock in DMSO) was added to 120 mm^3^ of MES buffer (50 mM, pH 7.5, containing 10 mM MgCl_2_, 1 mM EDTA, and 1 mM DTT). Subsequently, 10 mm^3^ of NADPH (100 mM in water), 50 mm^3^ of ATP (20 mM in water), and 60 mm^3^ of CAR enzyme preparation (4.5 mg/cm^3^ total protein, containing approximately 50 % of CAR) were added. The reaction proceeded in a Thermomixer at 28 °C and 300 rpm. After 2 h and overnight incubation, the reaction was acidified by the addition of aqueous HCl (4 N, 50 mm^3^) and the products were extracted with ethyl acetate (2 × 0.3 cm^3^). The extracts were dried over Na_2_SO_4_ and TMS-diazomethane solution (2 mm^3^, 2 M in diethyl ether) was added for the derivatization of **1a** to the corresponding methyl ester for GC analysis. GC–MS analyses were performed on an Agilent 7890A Series GC system equipped with a 5975C MS system, using a (5 % phenyl) methylpolysiloxane capillary column (HP-5MS, 30 m, 0.25, 0.25 µm film) with He as the carrier gas. The method was 100 °C hold 0.5 min, 10 °C/min to 300 °C. Retention times were: **1b**: 6.10 min; cinnamic alcohol (**1c**, a potential product derived from over-reduction): 6.47 min; methyl cinnamate (**1d**): 7.44 min.
